# Angiotensin peptide synthesis and cyclic nucleotide modulation in sympathetic stellate ganglia

**DOI:** 10.1016/j.yjmcc.2019.11.157

**Published:** 2020-01

**Authors:** Emma N. Bardsley, Oliver C. Neely, David J. Paterson

**Affiliations:** a Wellcome Trust OXION Initiative in Ion Channels and Disease, Oxford, UK; b Burdon Sanderson Cardiac Science Centre, Department of Physiology, Anatomy and Genetics, University of Oxford, Oxford OX1 3PT, UK; c British Heart Foundation, Centre of Research Excellence, UK

**Keywords:** Angiotensin, Sympathetic, Autonomic nervous system, Stellate ganglia, Hypertension

## Abstract

Chronically elevated angiotensin II is a widely-established contributor to hypertension and heart failure via its action on the kidneys and vasculature. It also augments the activity of peripheral sympathetic nerves through activation of presynaptic angiotensin II receptors, thus contributing to sympathetic over-activity. Although some cells can synthesise angiotensin II locally, it is not known if this machinery is present in neurons closely coupled to the heart. Using a combination of RNA sequencing and quantitative real-time polymerase chain reaction, we demonstrate evidence for a renin-angiotensin synthesis pathway within human and rat sympathetic stellate ganglia, where significant alterations were observed in the spontaneously hypertensive rat stellate ganglia compared with Wistar stellates. We also used Förster Resonance Energy Transfer to demonstrate that administration of angiotensin II and angiotensin 1–7 peptides significantly elevate cyclic guanosine monophosphate in the rat stellate ganglia. Whether the release of angiotensin peptides from the sympathetic stellate ganglia alters neurotransmission and/or exacerbates cardiac dysfunction in states associated with sympathetic over activity remains to be established.

## Highlights

• The stellate ganglia may act as a novel source for local angiotensin signaling.
• Angiotensin peptides and receptors were identified in human and rat stellates.
• Elevations in neuronal cGMP occur in response to angiotensin peptides.
• RNA transcript expression was altered in young spontaneously hypertensive rats.
• Angiotensin signaling at this site may be linked to dysautonomia in hypertension.

## 1 Introduction

The Renin-Angiotensin-Aldosterone system (RAAS) plays a central role in the physiological regulation of arterial blood pressure and fluid volume homeostasis [1]. Angiotensin II (AngII) is a major effector of the Renin-Angiotensin system (RAS) that mediates its effects in a wide range of organs [[2], [3], [4]] via selective activation of AngII receptors type 1 and 2 (AT_1_R, AT_2_R) [5,6]. Altered RAS signaling is evident in hypertension [[7], [8], [9], [10], [11]], heart failure [[12], [13], [14]] and post-myocardial infarction [[15], [16], [17], [18]], where AngII-AT_1_R activation is involved in enhanced sympathetic transmission [[19], [20], [21]], cardiac hypertrophy [8,13,[22], [23], [24]] impaired calcium handling [9], vascular remodeling [3,4] and pro-inflammatory events [25,26] that all contribute to cardiac arrhythmia [27].

Therapeutic inhibition of classical AngII signaling is an effective tool for the treatment and management of renal and cardiovascular diseases (CVDs), [[28], [29], [30], [31]] although the precise mechanism, site/s of action and source/s of angiotensins are not entirely clear [11,28,32,33]. Emerging evidence highlights a dominant role for local production rather than systemic angiotensin signaling at its sites of action, where key proteins are synthesized intracellularly acting in an autocrine and/or paracrine manner on local and neighboring tissues [2,3,34]. Whether the intracellular machinery is present and conserved in neurons that predominately innervate the heart is not known.

Immunocytological studies have highlighted the presence of Ang II-positive neurons within the coeliac sympathetic ganglia that innervate mesenteric blood vessels [35], the trigeminal [36] and dorsal root ganglia [37,38]. More recently, AngII immunoreactivity has been demonstrated within atrial nerve fibers, even though the derivation of these fibers is not firmly established [39]. In this study, we used a combination of RNA sequencing (RNA-seq), qRT-PCR and ELISA assays to demonstrate that the cervicothoracic sympathetic stellate ganglia (T1-T4) that predominantly innervate the heart, possess an intracrine RAS system that is conserved in both human and rat.

## 2 Materials and methods

### 2.1 Clinical samples

For clinical samples, human stellate ganglia were kindly obtained and sent by Dr. Ajijola, Dr. Ardell and Dr. Shivkumar from the UCLA Cardiac Arrhythmia Center. Briefly, both the right and left stellate ganglia were identified lying anterior to the neck of the first rib on each side, and following identification of the subclavian artery. Ganglia were dissected and removed in their entirety. Demographic information and disease history were obtained and the characteristics of human donors are included in Table 1. The human study was approved by the UCLA Institutional Review Board (approval # 12-000701) and informed consent was obtained from all subjects.

**Table 1:** Human stellate donor characteristics.

Donor	Gender	Age	Complications
#19	Male	77	Non-ischaemic cardiomyopathy, ventricular fibrillation, LVEF 30–35%
#21	Female	34	Excessive tachycardia, normal heart, LVEF presumed normal
#22	Male	70	Ischaemic cardiomyopathy, ventricular tachycardia, coronary artery disease, LVEF 30%
#23	Male	19	Healthy, normal heart size, and chamber, LVEF 55–60%
#24	Male	62	Non-ischaemic cardiomyopathy, polymorphic ventricular tachycardia/fibrillation, LVEF 30–50%

### 2.2 Animals

Three- to-four-week-old young male prehypertensive spontaneously hypertensive rats (preSHR), 12-to-16-week-old or 18-to-20-week-old adult male spontaneously hypertensive rats (SHR) with established hypertension and age-matched, male normotensive Wistar control rats were obtained from Envigo, UK. Details regarding the model are described in the supplementary file. All rats were housed in standard plastic cages and artificial lighting was fixed to a natural 12-hour light/dark cycle. Food and water were available ad libitum. All experiments were performed in accordance with the UK Home Office Animal Scientific Procedures Act 1986 (ASPA) and approved by the University of Oxford (PPL 30/3131).

### 2.3 Rat sympathetic cardiac ganglia dissection

Rats were anaesthetized in an induction chamber (3–5% isoflurane) and humanely killed by a Home Office approved Schedule 1 method: overdose of pentobarbital (Euthatal, 200 mg/ml) and exsanguination. Dissection was carried out as previously described [40].

### 2.4 RNA extraction from sympathetic cardiac ganglia

RNA was extracted from left and right sympathetic stellate ganglia from four-week-old and 16-week-old male SHR and age-matched male Wistar rats. Human stellate ganglia were shipped on dry ice in RNA*later*® RNA Stabilization Solution (ThermoFisher). Rat and human ganglia were cleaned and de-sheathed in dPBS without Ca^2+^ and Mg^2^ and RNA was extracted and quality assessed as previously described ^63^.

### 2.5 Library preparation for RNA sequencing

RNA extracted from the right or left stellate ganglia of four-week-old male preSHR (*n* = 5) and age-matched Wistar (n = 5) was sent to the High-Throughput Genomics Group at the Wellcome Trust Centre for Human Genetics (WTCHG) for RNA-seq library construction and sequencing using an Illumina HiSeq 4000 (Illumina, Inc., San Diego, USA). The sequencing libraries were amplified using a SMARTer (first-strand synthesis) amplification protocol and prepared for paired-end sequencing (2 × 75 bp). Each sample was sequenced on three separate lanes to minimize technical error and to increase the sequencing depth (~15–25 million reads per lane). Samples were randomized and blinded to the experimenter. The number of replicates and the sequencing parameters established, were based on recommendations from WTCHG and those published by Conesa et al., 2016 [41].

### 2.6 RNA sequencing analysis

Transcripts were quantified via the Salmon package (version 0.8.2) using the transcriptome-based quasi-mapping-based mode [42]. The data were imported into R and summarized at the gene-level using the ‘tximport’ function (v1.6.0) as per the vignette [43]. A differential expression analysis of the gene counts for Wistar and preSHR samples was performed using the ‘DESeq2’ command in the R package DESeq2 (v1.18.1) [44]. To assess the relevance of the observed differentially expressed genes, the significantly different transcripts at the Benjamini-Hochberg p. adj < 0.05 level were analyzed using the Database for Annotation, Visualization and Integrated Discovery (DAVID v6.8) tool suite [45]. A Kyoto Encyclopedia of Genes and Genomes (KEGG) [46] analysis was performed to provide information about pathway mapping. A full description of the RNA-seq analysis is included in the supplement.

### 2.7 Quantitative real-time polymerase chain reaction

50 ng of RNA was used to construct each cDNA library (*n* = 4/group). For rat ganglia, the SuperScript™ III VILO™ cDNA synthesis protocol was followed according to manufacturer's instructions (ThermoFisher). For human ganglia, the SuperScript™ IV VILO™ cDNA synthesis protocol was followed according to manufacturer's instructions (ThermoFisher) as previously described [40]. Concentrations of cDNA in each sample and the 260/280 ratios were calculated (NanoDrop Lite) to detect the presence of contaminants. Samples with an abnormal 260/280 ratio (<1.7 and >1.95) were discarded. Samples were aliquoted and frozen at −80 °C for long-term storage or kept refrigerated at 4 °C for immediate use.

### 2.8 Two-step qRT-PCR

Two-step qRT-PCR was used to confirm the presence of the following mRNA transcripts in the stellate ganglia cDNA libraries: angiotensinogen (AGT, *Agt;* Rn00593114_m1, Hs01586213_m1; rat, human respectively), renin (*Ren*; Rn00561847_m1, Hs00982555_m1; rat, human), angiotensin converting enzyme (ACE, *Ace,* Rn00561094_m1, Hs00174179_m1; rat, human), angiotensin converting enzyme type 2 (ACE2, *Ace2*; Rn01416293_m1, Hs01085333_m1; rat, human), angiotensin II receptor subtype 1a (AT_1A_R, *Agtr1a*; Rn02758772_s1; rat), angiotensin II receptor subtype 1b (AT_1B_R, *Agtr1b*; Rn02132799_s1; rat), angiotensin II receptor type 1 (AT_1_R, *Agtr1*; Hs00258938_m1; human), angiotensin II receptor type 2 (AT_2_R, *Agtr2*; Rn00560677_s1, Hs02621316_s1; rat, human), Mas receptor (Mas R, *Mas1*; Rn00562673_s1, Hs00267157_s1; rat, human). The following controls were selected given the similar levels of beta 2 microglobulin *(B2m)* expression in stellates from Wistar and SHR in the RNA-seq dataset: B_2_m (*B2m;* Rn00560865_m1, Hs00187842_m1; rat, human), glyceraldehyde-3-phosphate dehydrogenase (*Gapdh;* Rn99999916_s1, Hs02786624_g1; rat, human). TaqMan® probes were used to evaluate the expression of the genes of interest and qRT-PCRs were carried out as described in the supplement.

### 2.9 Förster resonance energy transfer (FRET)

For FRET measurements of cytosolic cGMP, sympathetic stellate neurons from four-week-old preSHR Wistar rats were cultured into a single-cell suspension using a previously described method [40] and transduced with the FRET biosensor cGi500 (3.42 × 10^8^ pfu/well, Vector BioLabs) in neuronal plating media. After 24-h, the virus-containing medium was replaced with virus-free neuronal plating medium and the neurons were incubated for a further 24–36 h (37 °C, 5% CO_2_) to obtain an appropriate level of biosensor expression for FRET imaging as previously described [40,47].

Sensor expressing stellate neurons were imaged on an inverted Nikon microscope connected to an OptoLED fluorescence imaging system (Cairn Research Ltd) as described in the data supplement. During FRET experiments, stellate neurons were perfused continuously with HEPES-buffered Tyrode's solution (in mM): 135 NaCl, 4.5 KCl, 11 glucose, 1 MgCl_2_, 2 CaCl_2_, 20 HEPES, adjusted to pH 7.4. Experiments were conducted at room temperature using a gravity-fed perfusion system and the flow rate was controlled at 2–3 ml/min. A stable baseline of at least 2 min was recorded at the start of each experiment. Randomly selected neurons expressing the FRET sensor from Wistar (*n* = 20 cells) or preSHR (*n* = 19 cells) were stimulated with AngII (1 μM) followed by Ang1–7 (1 μM) diluted in Tyrode's solution and the resulting FRET change was recorded for 5 min. In vehicle-controlled experiments, cells were exposed to Tyrode solution (*n* = 4). In all experiments, the maximal FRET change of each cell was recorded by exposing the cells to saturating concentrations of the NO donor Sin-1 (1 μM) and the PDE inhibitor IBMX (100 μM) to ensure that the cells expressing the sensor responded similarly. Cells that did not respond appropriately to the maximal test stimulation were removed from the final analysis. The FRET data were collected from each cell and averages were calculated over time. The peak FRET changes were calculated as the maximal response generated by AngII or Ang1-7. Peak responses were expressed as a proportion (%) of the maximally evoked FRET change with Sin-1 and IBMX.

### 2.10 Protein extraction and assay protocols

Protein was extracted from human stellate ganglia that were processed individually. The stellate samples were homogenized in ice-cold dPBS without Ca^2+^ or Mg^2+^ and the protein within the samples was normalized using a standard protein assay (BioRad DC). Enzyme immuno assays (EIA) or enzyme-linked immunosorbent assays (ELISA) were conducted to detect the presence of the following proteins of interest in human stellate ganglia: AGT (CSB-E08564h, Cusabio), renin (dren00, R&DSystems), AngII (RAB0010-1KT, Sigma), ACE2 (LS-F5886, LSBio), Ang1-7 (CSB-E14242h). Briefly, standards or samples (2–3 repeats) were incubated in 96-well plates and each assay was carried out as per the manufacturer's instructions. The absorbance or fluorescence from each well was measured within 5 min at the appropriate wavelength and the background was subtracted from primary absorbance values (Infinite F500, Tecan). The expression of each relevant protein was quantified using a standard curve generated from the supplied standards (GraphPad Prism, v7).

### 2.11 Statistical analysis

RNA-seq data were analyzed using the Salmon Quasi-mapping method in the statistical programme R, as described in the data supplement. The RNA-seq raw files are deposited in NCBI sequence read archive (SRA) under the accession PRJNA591289. Other data were imported into GraphPad Prism software (v7) for graphical representation. All data are expressed as mean ± SEM. The FRET data were analyzed using a two-way analysis of variance (ANOVA) and peak values analyzed using an unpaired two-tailed Student's *t*-test. All data are expressed as the mean ± SEM. Statistical significance was accepted at *p* < .05 unless otherwise described.

## 3 Results

### 3.1 Angiotensinergic mRNA transcript expression in human stellate ganglia

**Fig. 1:**
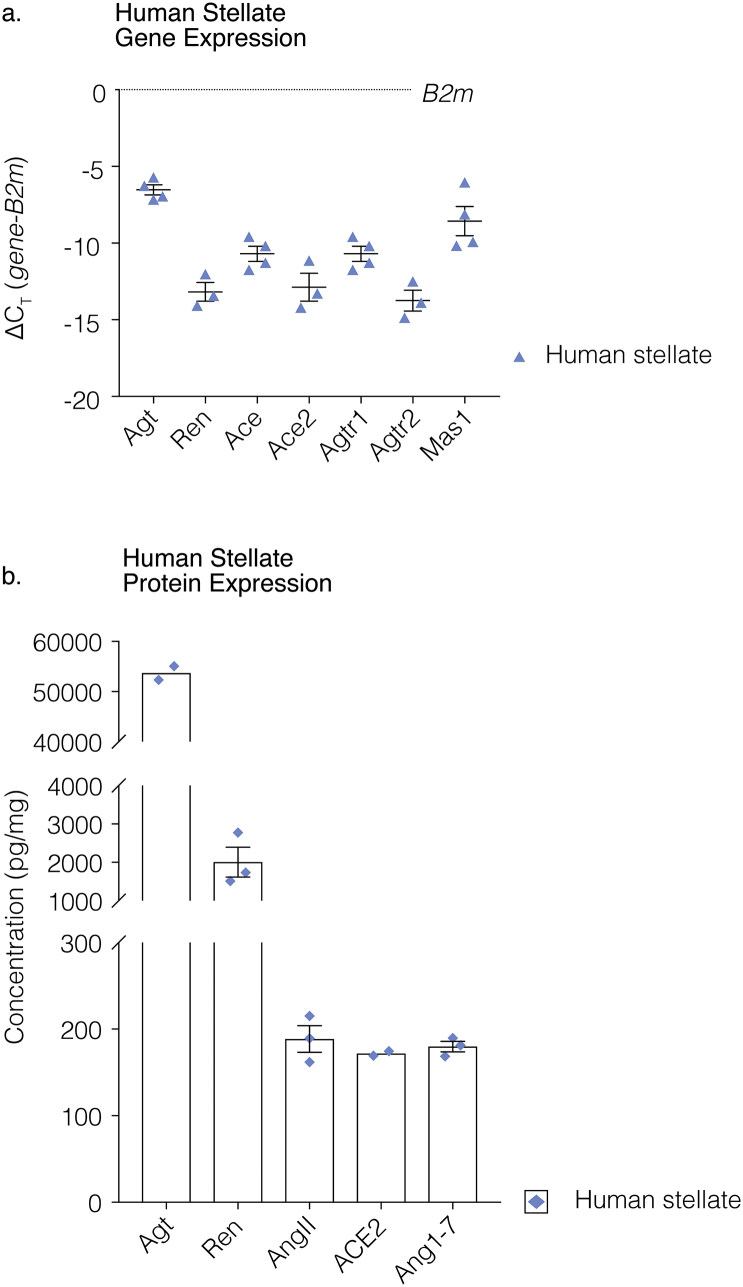
Angiotensin synthesizing enzymes and precursors are expressed in human stellate ganglia. In human stellate ganglia the presence of the mRNA transcripts encoding *Agt* (*n* = 4), *Ren* (*n* = 3)*, Ace* (n = 4)*, Ace2* (n = 3)*, Agtr1* (n = 4)*, Agtr2* (n = 3) and *Mas1* (n = 4) were confirmed by qRT-PCR. The qRT-PCR raw counts for the genes of interest were normalized to the control gene *B2m* using the ∆C_T_ method and expressed as ∆C_T_ mean ± SEM (a). ELISAs were used to demonstrate the protein expression of the relevant proteins of interest including Agt, Ren, AngII, ACE2 and Ang1–7 in human stellate ganglia. Agt was found to be highly expressed in human stellate ganglia (*n* = 2, ~53,694 pg/mg), as was Ren (n = 3, 2005 ± 388 pg/mg). AngII (n = 3, 188.7 ± 15.37 pg/mg), ACE2 (n = 2, 171.9 ± 2.60 pg/mg) and Ang1–7 (n = 3, 179.9 ± 6.13 pg/mg) were also identified and were found to have similar levels of expression (b). Data are displayed as mean ± SEM. A model diagram depicts AngII and Ang1–7 release from the stellate ganglia and the proposed pre-and post-synaptic effects.

In order to ascertain the translational relevance of our study, we sought to identify whether key mRNA transcripts were present in human stellate ganglia. We found the presence of the mRNA transcripts encoding *Agt* (*n* = 4), *Ren* (*n* = 3), *Ace* (*n* = 4), *Ace2* (*n* = 3), *Agtr1* (*n* = 4), *Agtr2* (*n* = 3) and *Mas1* (*n* = 4) were confirmed by qRT-PCR. The qRT-PCR raw counts for the genes of interest were normalized to the control gene *B2m*, using the ∆C_T_ method described by Schmittgen et al. [48], and expressed as normalized count values (Fig. 1A).

### 3.2 Angiotensin synthesizing enzymes and precursors are expressed in human stellate ganglia

ELISAs were used to demonstrate the expression of the relevant proteins of interest including Agt, Ren, AngII, ACE2 and Ang1-7 in human stellate ganglia. Agt was found to be highly expressed in human stellate ganglia (*n* = 2, ~53,694 pg/mg), as was Ren (*n* = 3, 2005 ± 388 pg/mg). AngII (*n* = 3, 188.7 ± 15.37 pg/mg), ACE2 (*n* = 2, 171.9 ± 2.60 pg/mg) and Ang1-7 (*n* = 3, 179.9 ± 6.13 pg/mg) (Fig. 1B). These data indicate that an angiotensinergic profile is also evident in the human sympathetic stellate ganglia.

### 3.3 RNA sequencing reveals altered pathways coupled to renin secretion in rat stellate ganglia

**Fig. 2:**
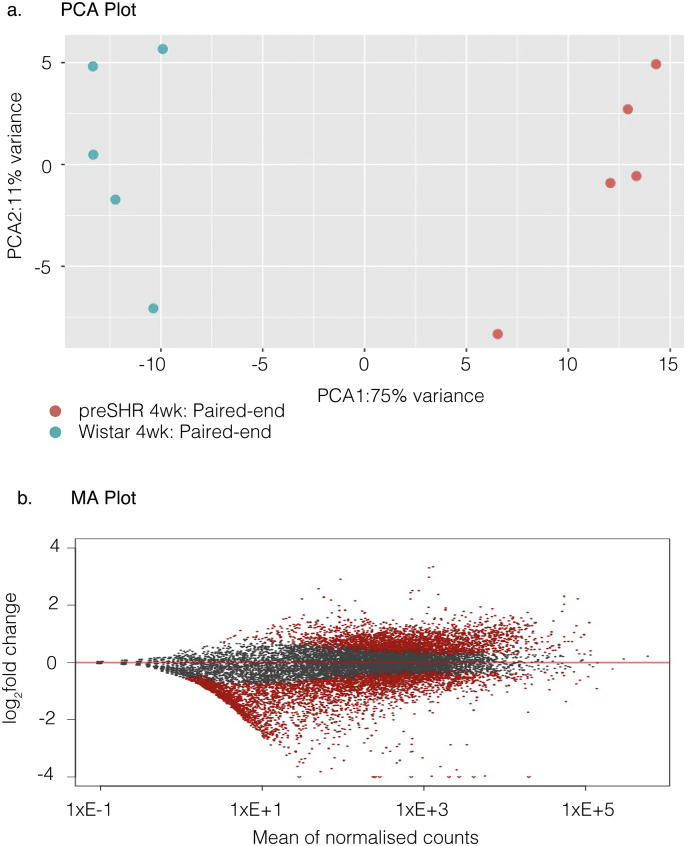
The principal component (PCA) plot illustrates the variation of the stellate ganglia transcriptome between strains (a). The MA plot depicts the relationship between the number of counts per gene, the size of the fold change and the significance of fold changes. Significance is indicated by red spheres on a gene-by-gene basis. Non-significant transcripts are shown in grey (b).

RNA-seq was carried out as a non-biased, hypothesis neutral approach to sequence the transcriptome of the sympathetic stellate ganglia. RNA was extracted from the stellate ganglia of young, four-week male Wistar (*n* = 5) and age-matched male, preSHR (*n* = 5). We obtained an average number of mapped reads (70.15% ± 1.658%) that was not significantly different between strains (Table 2). DESeq2 was used to generate a principal component analysis (PCA) plot with log_2_ normalization to demonstrate variation (Fig. 2A). An MA plot was generated for visual representation of the genomic data (Fig. 2B). The data were analyzed using the Salmon quasi-mapping tool [49] with functions applied to correct for GC and positional bias. The data were quantified into gene level expression values and DESeq2 [50] was used to calculate the differentially expressed mRNA transcripts between strains. In confirmation of the expected sympathetic neuronal phenotype, markers consistent with sympathetic neurons were among the most highly expressed genes in both strains (Table 3), including dopamine beta hydroxylase (*Dbh*), neuropeptide Y (*Npy*), and tyrosine hydroxylase (*Th*).

**Table 2:** RNA-seq Mapping rates.

Strain	Age	Total reads	Mapping rate
Wistar	4 wk	18,230,963	63.26%
Wistar	4 wk	19,805,596	70.84%
Wistar	4 wk	16,568,807	61.53%
Wistar	4 wk	18,609,695	66.86%
Wistar	4 wk	21,109,646	76.57%
preSHR	4 wk	20,929,135	72.21%
preSHR	4 wk	19,893,408	71.92%
preSHR	4 wk	21,087,019	72.14%
preSHR	4 wk	20,480,603	68.35%
preSHR	4 wk	22,476,940	77.81%

The RNA-seq mapping rates indicate the number of assigned reads relative to the total number of reads in 4-week-old, male, Wistar and prehypertensive SHR stellate ganglia. The total number of reads is also shown. LS, left stellate; RS, right stellate.

**Table 3:** Highest abundant transcripts in young Wistar and preSHR stellate ganglia.

	Wistar stellate			PreSHR stellate		
	Gene	Gene name	Count	Gene	Gene name	Count
1	*Tuba1a*	Tubulin, Alpha 1A	*566,177.2*	*Tuba1a*	Tubulin, Alpha 1A	*622,220*
2	*Ubb*	Ubiquitin B	*297,045.6*	*Ubb*	Ubiquitin B	*302,595*
3	*Tubb3*	Tubulin Beta 3 Class III	*245,554.2*	*Tubb3*	Tubulin Beta 3 Class III	*194,890*
4	***Dbh***	**Dopamine beta hydroxylase**	*190,251.4*	*Tmsb4x*	Thymosin Beta 4 X-Linked	*160,003*
5	*Actg1*	Actin Gamma 1	*146,630.2*	*Actg1*	Actin Gamma 1	*137,129*
6	*Hspa8*	Heat Shock Protein Family A (Hsp70) Member 8	*135,640*	*Stau2*	Staufen Double-Stranded RNA Binding Protein 2	*133,080*
7	*Thy1*	Thy-1 Membrane Glycoprotein	*133,169.4*	***Npy***	**Neuropeptide Y**	*129,720*
8	*Aldoa*	Aldolase, Fructose-Bisphosphate A	*120,785.1*	*Ppia*	Peptidylprolyl Isomerase A	*125,088*
9	*Actb*	Beta-Actin	*110,882.4*	*Tuba1b*	Tubulin Alpha 1b	*123,908*
10	*Tuba1b*	Tubulin Alpha 1b	*109,086.1*	*Hspa8*	Heat Shock Protein Family A (Hsp70) Member 8	*115,132*
11	*Ndrg4*	NDRG Family Member 4	*102,766.3*	*Sncg*	Synuclein Gamma	*112,531*
12	***Npy***	**Neuropeptide Y**	*102,132.6*	*Tmsb10*	Thymosin Beta 10	*108,398*
13	*Gapdh*	Glyceraldehyde 3-phosphate dehydrogenase	*92,503.9*	*Aldoa*	Aldolase, Fructose-Bisphosphate A	*106,952*
14	***Stmn2***	**Superior Cervical Ganglion-10 Protein**	*90,422.6*	***Stmn2***	**Superior Cervical Ganglion-10 Protein**	*105,069*
15	*Hsp90ab1*	Heat Shock Protein 90 Alpha Family Class B Member 1	*89,436.4*	*Sepw1*	Selenoprotein W	*95,857.8*
16	*Ubc*	Ubiquitin C	*88,494.8*	*Gapdh*	Glyceraldehyde 3-phosphate dehydrogenase	*92,609.7*
17	*LOC310926*	Hypothetical protein	*83,996.98*	***Dbh***	**Dopamine beta hydroxylase**	*92,136.2*
18	*Uchl1*	Ubiquitin C-Terminal Hydrolase L1	*77,719.2*	*Rims3*	Regulating Synaptic Membrane Exocytosis 3	*91,409.1*
19	*Tubb5*	Tubulin Beta-5 Chain	*71,426.6*	*Uchl1*	Ubiquitin C-Terminal Hydrolase L1	*90,749.2*
20	*Eef1a1*	Eukaryotic Translation Elongation Factor 1 Alpha 1	*68,436.2*	*Eef1a1*	Eukaryotic Translation Elongation Factor 1 Alpha 1	*88,865*
21	*Zwint*	ZW10 Interacting Kinetochore Protein	*66,713.6*	*S100a6*	S100 Calcium Binding Protein A6	*88,557.8*
22	*Ppia*	Peptidylprolyl Isomerase A	*66,229*	*Hsp90ab1*	Heat Shock Protein 90 Alpha Family Class B Member 1	*88,011.8*
23	***Snap25***	**Synaptosome Associated Protein 25**	*65,940.7*	*Thy1*	Thy-1 Membrane Glycoprotein	*87,061.6*
24	***Th***	**Tyrosine hydroxylase**	*63,260*	*Sparc*	Secreted Protein Acidic And Cysteine Rich	*84,132.4*
25	*Sncg*	Synuclein Gamma	*62,918.8*	*Rps14*	Ribosomal Protein S14	*80,267.4*
26	*Eno1*	Enolase 1	*61,555.62*	*Calm2*	Calmodulin 2	*75,396.6*
27	*Pkm*	Pyruvate Kinase M1/2	*61,313*	*LOC257642*	rRNA promoter binding protein (provisional)	*70,419.3*
28	*Tmsb4x*	Thymosin Beta 4 X-Linked	*59,318.4*	*Gnas*	guanine nucleotide binding protein, alpha stimulating	*67,951.4*
29	*Fth1*	Ferritin heavy chain 1	*55,496*	*Actb*	Beta-Actin	*63,737.3*
30	*Ap2m1*	Adaptor Related Protein Complex 2 Subunit Mu 1	*54,113.4*	*LOC310926*	hypothetical protein	*62,149.5*

The 30 most abundantly expressed genes in stellate ganglia obtained from young four-week-old male Wistar and preSHR rats in the RNA-seq dataset. The estimated number of reads of each transcript are expressed as mean counts. Neuronal sympathetic markers are highlighted in bold. No significant differences were found in sympathetic markers between strains.

**Fig. 3:**
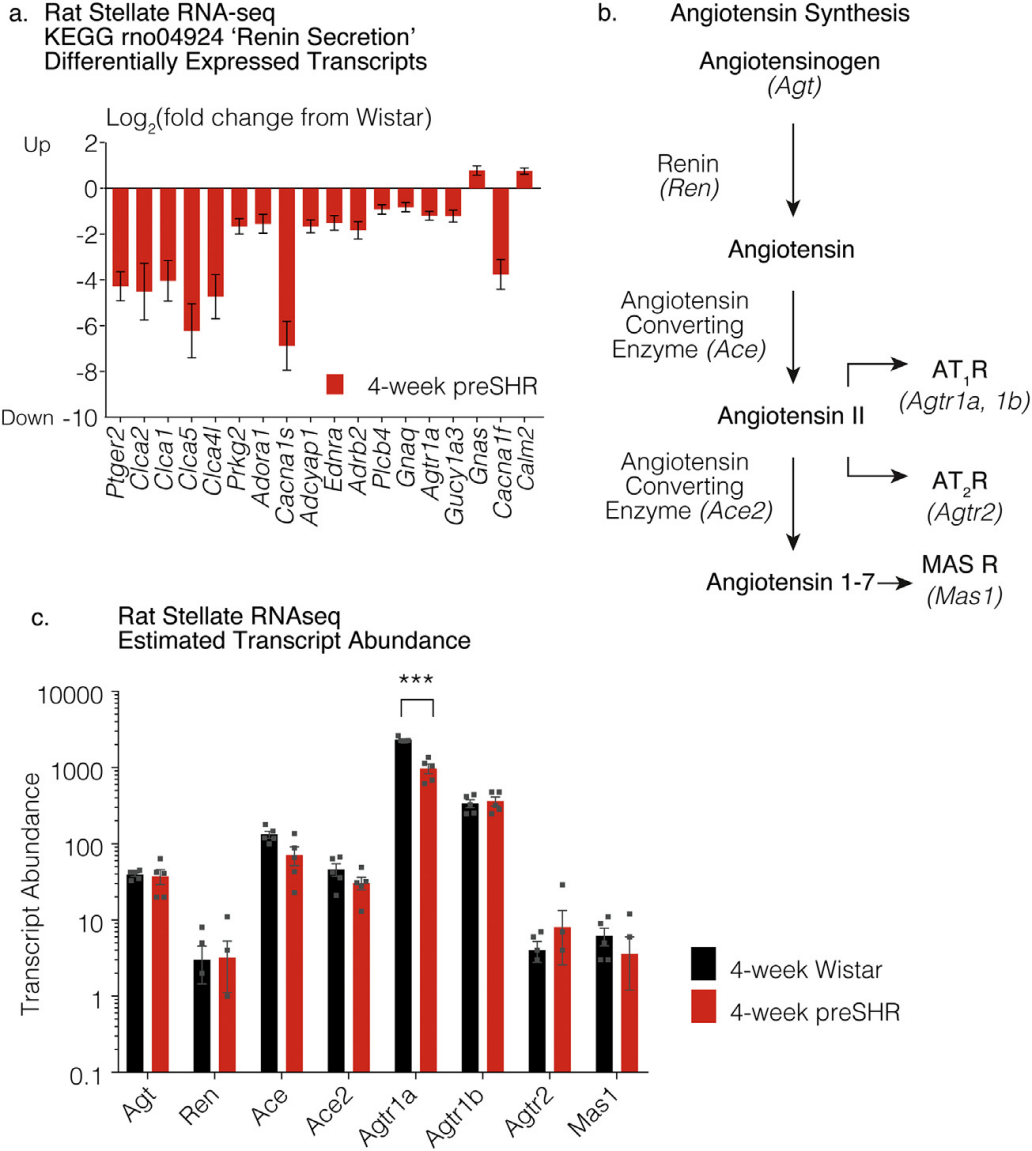
Transcripts of angiotensin synthesizing genes were observed in the rat sympathetic stellate ganglia in the RNA-seq dataset. The transcriptome of the sympathetic stellate ganglia was sequenced using stellate ganglia extracted from four-week-old male Wistar rats (*n* = 5) and age-matched male prehypertensive SHR (preSHR, n = 5). A KEGG analysis was carried out using the differentially expressed transcripts where the gene input was selected using the Benjamini-Hochburg p.adj < 0.05. The KEGG group ‘Renin Secretion’ was found to be significantly altered in the preSHR ganglia, where the gene input was selected using the Benjamini-Hochburg p.adj < 0.05 (a). A full list of the genes, the fold changes and respective levels of significance are reported in Table 4. The AngII and Ang1–7 synthesis pathways are outlined (b). Transcripts encoding the enzymes and precursors classically involved in the synthesis of AngII and Ang1–7 were identified in young rat stellate ganglia (b), where the relevant transcripts included Angiotensinogen (*Agt),* Renin (*Ren*) and the Angiotensin Converting Enzymes (*Ace, Ace2*). The transcripts for AngII receptors type 1 and 2 (*Agtr1a, Agtr1b,* Agtr2) and for the Ang1–7 receptor Mas (*M*as1*)* were also observed (c). Transcript abundances were not found to be differentially expressed in preSHR vs. Wistar ganglia, with the exception of *Agtr1a* that was significantly downregulated in the preSHR stellate ganglia (p. adj = 3.72 × 10^−8^, Salmon-DESeq2 method [85,86]).

To obtain information regarding the intracellular pathway enrichment, the differentially expressed genes selected at the Benjamini-Hochburg p.adjusted (p.adj) < 0.05 level were imported into the Database for Annotation, Visualization and Integrated Discovery (DAVID, v6.8) [45] and grouped using the Kyoto Encyclopedia of Genes and Genome (KEGG) function [46]. The KEGG group ‘Renin Secretion’ (rno04924) was found to be significantly different in the preSHR ganglia (*p* < .05), after a modified Fisher Exact statistical test was applied (*p* < .01) [45]. There were 18 differentially expressed genes observed in the KEGG category ‘Renin Secretion’ (rno04924) including *Ptger2, Clca2, Clca1, Clca5, Clca4l, Prkg2, Adora1, Cacna1s, Adcyap1, Ednra, Adrb2, Plcb4, Gnaq, Agtr1a, Gucy1a3, Gnas, Cacna1f, Calm2* (Fig. 3A). A list of the gene names, respective fold changes and levels of significance are reported in Table 4.

**Table 4:** Differentially expressed genes in the KEGG group ‘Renin Secretion’ (rno04924).

a				
Category	Term	Count	Genes	*P* value
rno04924	Renin secretion	18	*Ptger2, Clca2, Clca1, Clca5, Clca4l, Prkg2, Adora1, Cacna1s, Adcyap1, Ednra, Adrb2, Plcb4, Gnaq, Agtr1a, Gucy1a3, Gnas, Cacna1f, Calm2*	4.06E-02


b				
Gene name	Description	Count	Log_2_ fold change	*P*. adjusted
*Ptger2*	Prostaglandin E receptor 2	20.75	−4.28	7.01E-10
*Cacna1s*	Calcium voltage-gated channel subunit alpha1 S	11.02	−6.88	4.28E-09
*Agtr1a*	Angiotensin II receptor, type 1a	1610.44	−1.2	3.72E-08
*Adcyap1*	Adenylate cyclase activating polypeptide 1	144.68	−1.66	1.03E-07
*Cacna1f*	Calcium voltage-gated channel subunit alpha1 F	16.48	−3.76	1.52E-07
*Calm2*	Calmodulin 2	61,404.61	0.75	7.48E-07
*Clca5*	Chloride channel calcium activated 5	6.99	−6.22	2.18E-06
*Prkg2*	Protein kinase, cGMP-dependent, type II	241.03	−1.66	9.76E-06
*Clca4l*	Chloride channel calcium activated 4-like	11.38	−4.73	1.22E-05
*Adrb2*	Adrenoceptor beta 2	111.04	−1.84	1.29E-05
*Ednra*	Endothelin receptor type A	61.12	−1.51	2.27E-05
*Gucy1a3*	Guanylate cyclase 1 soluble subunit alpha 3	1078.49	−1.22	3.48E-05
*Plcb4*	Phospholipase C, beta 4	5858.51	−0.92	4.97E-05
*Clca1*	Chloride channel calcium activated 1	10.62	−4.04	5.24E-05
*Gnaq*	G protein subunit alpha q	223.72	−0.83	2.45E-04
*Gnas*	G Protein Subunit Alpha S	55,233.54	0.77	9.20E-04
*Adora1*	Adenosine A1 receptor	106.96	−1.55	1.04E-03
*Clca2*	Chloride channel calcium activated 2	4.96	−4.51	1.46E-03

The KEGG functional enrichment pathway representing ‘Renin Secretion’ (rno04924) was significantly over-represented in the SHR ganglia in the RNA-seq data, based on a significance at p < .05 (a). Eighteen genes within the KEGG group representing ‘Renin Secretion’ (rno04924) were downregulated. The log fold changes of the expression of each gene in the preSHR stellate ganglia and the Benjamini-Hochburg p.adj values are shown (b). The analysis was conducted in DAVID v6.8.

### 3.4 RNA sequencing reveals transcripts encoding angiotensin precursors and synthesizing genes in the rat sympathetic stellate ganglia

The RNA-seq dataset highlighted the presence of mRNA transcripts involved in the synthesis of AngII including *Agt*, *Ren*, *Ace* and angiotensin converting enzyme 2 (*Ace2),* responsible for Angiotensin 1-7 (Ang1-7) synthesis, [51,52]. We also identified the presence of the AngII receptor transcripts AT_1A_R, AT_1B_R and AT_2_R (*Agtr1a, Agtr1b, Agtr2*)*.* The transcript for the Ang1-7 receptor Mas (*Mas1)* was also identified in these ganglia (Fig. 3B–C).

### 3.5 Angiotensinergic mRNA transcript validation by qRT-PCR in rat stellate ganglia

**Fig. 4:**
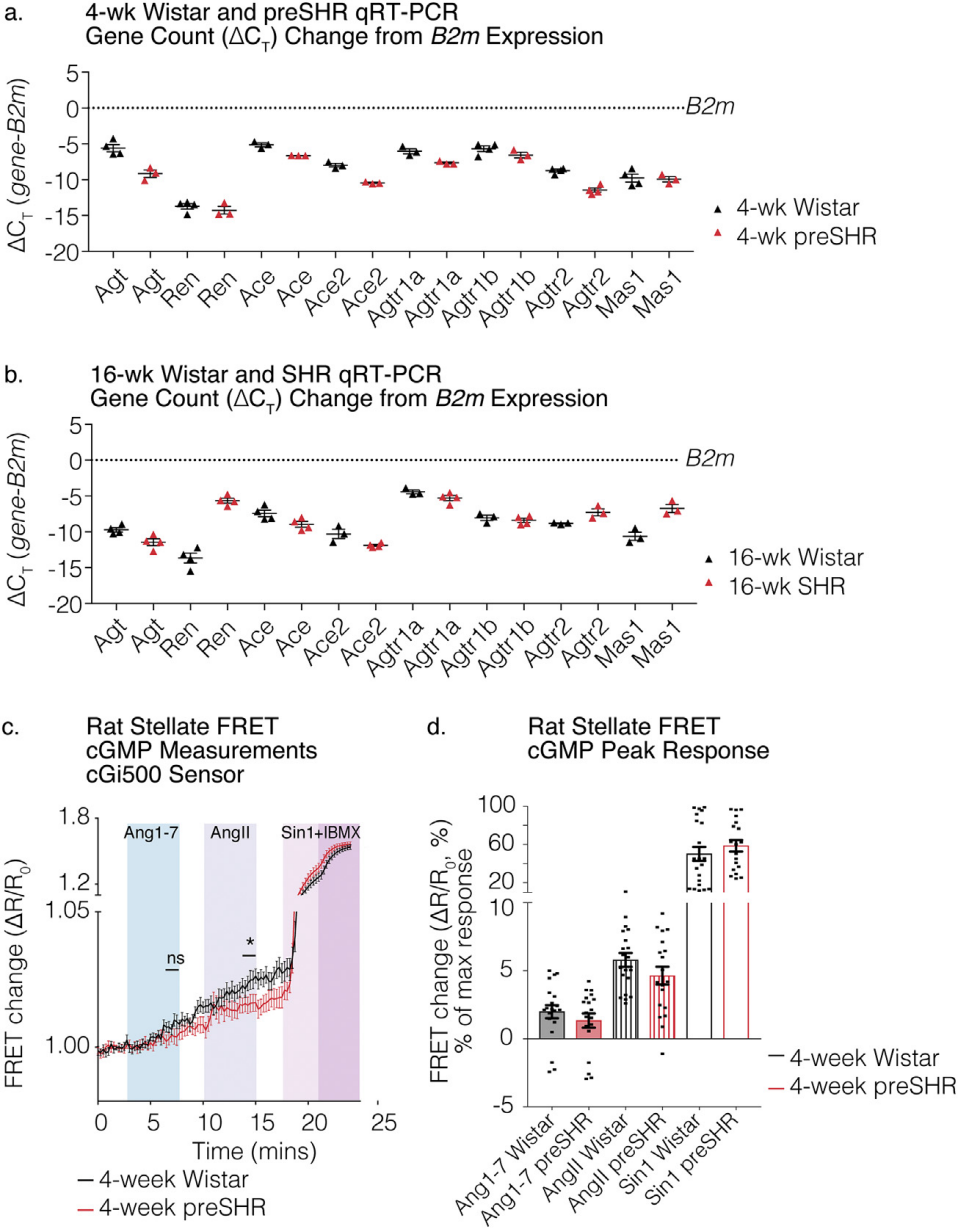
Angiotensinergic mRNA transcript validation by qRT-PCR in rat stellate ganglia. The presence of the RNA transcripts *Agt, Ren, Ace, Ace2, Agtr1a, Agtr1b, Agtr2* and *Mas1* was confirmed by qRT-PCR in sympathetic stellate ganglia from four-week Wistar and preSHR ganglia (a), and 16-week adult Wistar and SHR (b). The qRT-PCR raw counts were first normalized to a control gene *B2m* as per the comparative (∆C_T_) method [48]. Each data point corresponds to one stellate RNA sample from one rat. Data are displayed as ∆C_T_ mean ± SEM. FRET microscopy was conducted on sympathetic stellate neurons obtained from Wistar (*n* = 11 rats, 3 cultures, 20 cells) and preSHR rats (*n* = 9 rats, 3 cultures, 19 cells). Cells were transduced with the cGi500 FRET sensor and randomly selected for imaging. Increases in cGMP generation was observed in sympathetic neurons in response to Ang1–7 and AngII (c, d). Maximal FRET changes were evoked following administration of a combination of the NO-donor Sin-1 (10 μM) and the PDE inhibitor IBMX (100 μM). There was significantly greater cGMP generation in response to AngII in Wistar vs. preSHR neurons (two-way ANOVA, *p* = .0403). Peak FRET changes were obtained in response to AngII or Ang1–7 and converted to percentage FRET changes and values are depicted as a proportion of the maximal FRET change (%). There was no difference in peak FRET responses in response to Ang1–7 or between strains (d). Data are displayed as mean ± SEM.

RNA was extracted from the sympathetic stellate ganglia from male, four-week-old, young Wistar rats (*n* = 3–4 rats), young preSHR (*n* = 3–4 rats), 16-week adult SHR (*n* = 4–5 rats), and age-matched adult Wistar rats (*n* = 4–5 rats). Quantitative real-time polymerase chain reactions (qRT-PCR) were performed to validate the presence and relative levels of the mRNA transcripts encoding AngII and Ang1-7 synthesizing enzymes and precursors (*Agt, Ren, Ace, Ace2*), in addition to their respective receptor targets (*Agtr1a, Agtr1b, Agtr2, Mas1*) in four-week and 16-week rat stellate ganglia (Fig. 4A-C). In the four-week Wistar and preSHR ganglia, we confirmed presence of the mRNA transcripts encoding *Agt* (*n* = 4,3 Wistar, preSHR respectively) *Ren* (n = 4,3)*, Ace* (*n* = 3,3)*, Ace2* (n = 3,3) *Agtr1a* (n = 3, 3)*, Agtr1b* (n = 4, 3)*, Agtr2* (n = 4, 4)*,* and *Mas1* (n = 4, 3). In the 16-week adult Wistar and SHR ganglia, qRT-PCRconfirmed the presence of the mRNA transcripts encoding *Agt* (n = 4,4 Wistar, preSHR respectively) *Ren* (n = 4,4)*, Ace* (n = 4,4)*, Ace2* (n = 3,4) *Agtr1a* (n = 3, 4)*, Agtr1b* (n = 3, 4)*, Agtr2* (n = 3, 3)*,* and *Mas1* (n = 3, 3). Technical replicates and subsequently biological replicates were averaged. Raw gene counts were normalized to a control gene *B2m* and the ∆C_T_ was calculated as per the method described by Schmittgen et al. [48]. Together, these data highlight an angiotensinergic presence in the sympathetic stellate ganglia of rat.

### 3.6 cGMP generation by angiotensin peptides

**Fig. 5:**
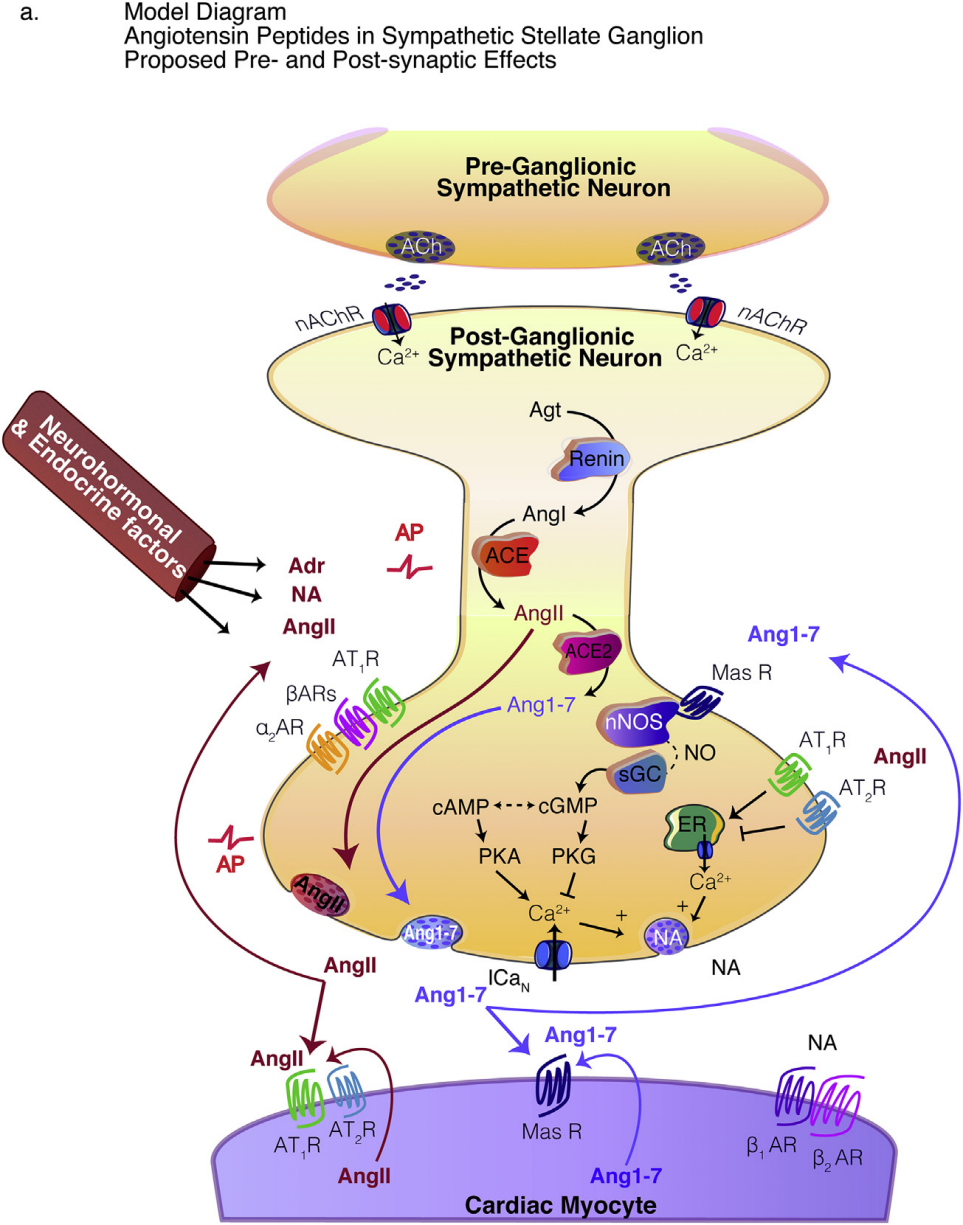
Model diagram depicts angiotensin synthesis and pre- and post-synaptic signaling pathways. In sympathetic stellate neurons, the classical pathway for Angiotensin II (AngII) synthesis occurs by sequential enzymatic cleavage of Angiotensinogen (Agt) by renin and Angiotensin Converting Enzymes (ACE). AngII is hydrolyzed by ACE2 producing the bioactive metabolite of Angiotensin 1–7 (Ang1–7). We identified the presence of precursors and transcripts encoding these enzymes and depict here a proposed model for angiotensin synthesis (a). We also identified the presence of AngII and Ang1–7 receptors on sympathetic stellate ganglia of human and rat. AngII has been shown to elevate intracellular Ca^2+^ and enhance noradrenaline release via actions at AT_1_R [59,60]. Conversely Ang1–7-dependent activation of its cognate receptor, Mas R, has been shown to couple to NO in the brain and several other receptor sites [61]. In this study, we show that administration of both AngII and Ang1–7 elevate cGMP in the rat stellate ganglia. We and others have previously demonstrated the importance of NO-cGMP signaling in reducing [Ca^2+^]_i_ [47,63] and end-organ transmission in peripheral sympathetic stellate nerves [47,53,64,65] although the effects of Ang1–7 may be biphasic [66]. Dotted lines indicate intermediates in these intracellular signaling pathway. Several effects of AngII and Ang1–7 on the myocardium have been established [22,[67], [68], [69], [70], [71]].

The receptors AT_2_R and Mas R have been previously shown to couple to nitric oxide (NO)-cyclic guanosine monophosphate (cGMP) signaling pathways. Following the observation of *Agtr2* and *Mas1* in samples obtained from rat stellate ganglia, we aimed to investigate whether peptide activation of these receptors on cultured rat stellate neurons led to detectable increases in cGMP, using Förster Resonance Energy Transfer (FRET). Sympathetic stellate neurons from young, four-week-old male Wistar and age-matched male preSHR were transduced with the cGMP FRET sensor cGi500 and the resulting FRET changes were measured in response to AngII (1 μM) or Ang1–7 administration (1 μM). It was found that both Ang1–7 and AngII facilitated cGMP generation in Wistar (*n* = 20) and preSHR (*n* = 19) cells (Fig. 4C-D). Moreover, AngII led to a small but significant increase in cGMP generation in Wistar versus preSHR stellate neurons (two-way repeated measures ANOVA, *p* = .0403), however there was no measurable difference in the responses to Ang1–7 between strains. Maximal FRET change was achieved with a combination of the NO-donor Sin-1 (10 μM) and the non-selective PDE inhibitor IBMX (100 μM). The peak responses to AngII or Ang1–7 are shown as a proportion of the sensor's maximal response (Fig. 4D). No changes in fluorescence were observed in vehicle-controlled experiments during Tyrode administration (supplement Fig. 3A). A model diagram highlighting key angiotensin synthesis and receptor-coupled signaling pathways are depicted in our model diagram (Fig. 5).

## 4 Discussion

In this study we report several novel findings. First, we demonstrate that the genes involved in the synthesis of AngII are present in both human and rat stellate ganglia indicating that an intracellular RAS signaling system exists and is conserved across species within these neurons. Secondly, we confirm that AngII precursor peptides, AngII-synthesizing enzymes, and AngII itself, are present in human stellate ganglia. Thirdly, we document the presence of the bioactive AngII-metabolite Ang1-7 in human stellate ganglia and show evidence for its cognate receptor Mas R within this neuronal site. Finally, we used FRET to measure increases in cGMP generation in response to AngII and Ang1-7 suggesting a feedback modulatory role exists for angiotensin peptides within the sympathetic stellate ganglia.

We carried out an RNA-seq analysis to obtain an overview of the transcriptome of the stellate ganglia from young four-week preSHR and age-matched Wistar rats. The functional enrichment pathway analysis highlighted an alteration in the KEGG group ‘Renin Secretion’ (rno04924) in SHR stellates and a comprehensive search within the RNA-seq dataset subsequently highlighted the expression of all relevant gene transcripts involved in angiotensin peptide synthesis in the rat cardiac stellate ganglia (Fig. 3). We also confirmed the expression of these gene transcripts using qRT-PCR in stellate samples obtained from young four-week-old and adult 16-week-old SHR and Wistar rats. We measured a significant downregulation in *Agt*, *Ace* and *Ace2* transcript expression in the SHR strain at four and 16-weeks of age relative to controls. These data suggest that perturbations in AngII synthesis may occur alongside sympathetic dysfunction in this strain, where characteristics of sympathetic dysfunction include elevated intracellular Ca^2+^ [53,54], increased neuronal firing frequency [55], enhanced noradrenaline release [56,57] and alterations in neurotransmitter profiles [40,58].

We identified the presence of AngII and Ang1-7 receptors on sympathetic stellate ganglia of human and rat. AngII has been shown to elevate intracellular Ca^2+^ and enhance noradrenaline release via actions at AT_1_R [59,60], whereas activation of the Ang1-7 receptor, Mas R, has been shown to couple to NO in the brain and other receptor sites [61,62]. In this study, we show that administration of both AngII and Ang1-7 elevate cGMP generation in the rat stellate ganglia, suggesting a modulatory role for angiotensinergic peptides. We and others have previously demonstrated the importance of NO-cGMP signaling in reducing [Ca^2+^]_i_ [47,63] and end-organ transmission in peripheral sympathetic stellate nerves [47,53,64,65], although the effects of Ang1-7 may be biphasic [66]. Several effects of AngII and Ang1-7 on the myocardium have been previously established [22,[67], [68], [69], [70], [71]], however, our model figure depicts a proposed hypothesis for both the pre- and post-synaptic effects of AngII and Ang1-7 in the cardiac-sympathetic stellate ganglia. Moreover, alterations in RAAS signaling within specific sites precede increases in blood pressure in the SHR suggesting this is an early cellular marker associated with sympathetic impairment [[72], [73], [74]].

In our study, human sympathetic stellate ganglia expressed key genes and proteins required for AngII synthesis. We confirmed the presence of AngII in human stellate ganglia supporting immunocytological evidence from Bohlender et al., who recently demonstrated angiotensinergic innervation of the human right atrium, although the source of the neuronal fibers was not clear [39]. We also show evidence for neuronal Ang1-7 within human stellates, supporting the evidence that this bioactive peptide plays a role alongside AngII in modulating cardiac excitability through elevations in NO [61,75]. The beneficial effects of myocardial NOcGMP signaling are well-established, where increases in NO-cGMP reduce intracellular Ca^2+^ via inhibition of the L-type Ca^2+^ channel and reuptake into sarcoplasmic reticulum stores [[76], [77], [78]]. Of interest, the receptor transcript for Ang1–7 (*Mas1*) was present within stellate ganglia of human and rat alongside the expression of previously reported classical AngII receptors (AT_1_R, AT_2_R). Considering the effects of AngII and Ang1–7 on cGMP generation, there exists a conceivable role for these peptides in presynaptic sympathetic nerve modulation [79,80]. Given that plasma and tissue levels of AngII are significantly altered in hypertension [7,8,10,11,81] this source of RAS may provide a novel opportunity for precise therapeutic targeting of sympathetic nerves, to attenuate the effects of AngII within the myocardium [3,61,82,83].

### 4.1 Limitations

In this study, we carried out a hypothesis neutral, non-biased approach to sequencing the transcriptome of sympathetic stellate neurons of four-week-old preSHR and age-matched normotensive Wistar rats. There are several limitations to this approach. First, the stellate ganglion comprises a heterogeneous population of neuronal and supporting cells including fibroblasts and astrocytes, where we noted the presence of vimentin and glial fibrillary acidic protein (GFAP) respectively, however; a high number of identified transcripts were of neuronal phenotype. Secondly, to assess the pathways involved in AngII and Ang1-7 synthesis, the presence of the classical ACE-ACE2 pathway was explored. Recent literature has also highlighted the importance of alternative enzymatic pathways that may be involved in AngII synthesis and breakdown, including neprilysin, endopeptidases and other metalloproteases [84] whose transcripts were also identified in the RNA-seq dataset. Additionally, angiotensinergic peptides have been identified in neurons and glial cells in the brain; where expression in both cell types has been shown to regulate resting arterial pressure [61]. In this study, the precise cell-type/s responsible for angiotensin production within the stellate ganglia are not known; however, the angiotensin genes were observed in the RNA-seq dataset alongside high abundance transcripts specific to sympathetic nerves, suggesting that the primary cell type within these ganglia is of a sympathetic phenotype.

## 5 Conclusion

Our data here demonstrate that sympathetic stellate ganglia may act as a novel source for local AngII and Ang1-7 production to target pre- and post-synaptic cardiac excitability. Here, we describe the presence of genes and proteins involved in the synthesis of AngII and its metabolite Ang1-7, in addition to their receptor partners on sympathetic stellate ganglia from human and rat. We also demonstrate that alterations in RAS transcripts are evident in young prehypertensive SHR prior to the onset of hypertension, which may result in altered control of cardiac excitability. As such, these results may have implications for the pathogenesis and progression of dysautonomia and associated CVDs.

## Declaration of Competing Interest

None.

## Appendix A Supplementary data

Supplementary material Image 1
