# Supplementary material for: Angiotensin peptide synthesis and cyclic nucleotide modulation in sympathetic stellate ganglia
Source: J Mol Cell Cardiol. 2020 Jan;138:234–43. doi: 10.1016/j.yjmcc.2019.11.157 (PMC7049903; doi:10.1016/j.yjmcc.2019.11.157)
Supplement: Supplementary file 1 — Supplementary material [file mmc1.docx]

Data Supplement

ANGIOTENSIN PEPTIDE SYNTHESIS AND CYCLIC NUCLEOTIDE MODULATION IN SYMPATHETIC STELLATE GANGLIA

E. N. Bardsley*^1,2^, O. C, Neely^2^, D. J. Paterson*^1,2^

^1^ Wellcome Trust OXION Initiative in Ion Channels and Disease, Oxford, UK;

^2^ Burdon Sanderson Cardiac Science Centre, Department of Physiology, Anatomy and Genetics, University of Oxford, Oxford, OX1 3PT, UK

***Corresponding Authors:** Dr. Emma N. Bardsley, University of Oxford, Department of Physiology, Anatomy and Genetics, Oxford, OX1 3PT, UK; emmabardsley91@gmail.com, tel. +64 (0) 220 102602. Prof. David J. Paterson, University of Oxford, Department of Physiology, Anatomy and Genetics, Oxford, OX1 3PT, UK; [david.paterson@dpag.ox.ac.uk](mailto:david.paterson@dpag.ox.ac.uk), tel. +44 (0)1865 272471.

Key Words: Angiotensin; Sympathetic; Autonomic Nervous System; Stellate ganglia; Hypertension.

1. **Materials and Methods**
   1. **Animals**

Three- to-four-week-old young male prehypertensive spontaneously hypertensive rats (preSHR), 12-to-16-week-old or 18-to-20-week-old adult male spontaneously hypertensive rats (SHR) with established hypertension and age-matched, male normotensive Wistar control rats were obtained from Envigo, UK. The SHR strain displays normal blood pressure at four-weeks of age, where increases in arterial blood pressure develop progressively from five- to- six-weeks of age[1-5]. The SHR displays established hypertension from 12-weeks onwards[4,6-12], therefore, where no significant differences were observed between the adult rat samples over 12-weeks of age, data were combined for analysis. We used the Wistar rat strain as the normotensive control, given that Wistar rats are the progenitor breeding strain for the Wistar-Kyoto (WKY) and the two strains display similar hemodynamic profiles at all ages[3,5,10,13-15]. Additionally, neither strain display elevated sympathetic Ca^2+^ as observed in the SHR model[16] making the Wistar a suitable control in this study.

- 1. **Library preparation for RNA Sequencing**

RNA extracted from the right or left stellate ganglia of four-week-old male preSHR (n=5) and age-matched Wistar (n=5) was sent to the High-Throughput Genomics Group at the Wellcome Trust Centre for Human Genetics (WTCHG) for RNA-seq library construction and sequencing using an Illumina HiSeq 4000 (Illumina, Inc., San Diego, USA). The sequencing libraries were amplified using a SMARTer (first-strand synthesis) amplification protocol and prepared for paired-end sequencing (2 x 75 bp). Each sample was sequenced on three separate lanes to minimize technical error and to increase the sequencing depth (~15-25 million reads per lane). Samples were randomized and blinded to the experimenter. The number of replicates and the sequencing parameters established, were based on recommendations from WTCHG and those published by Conesa et al., 2016[17].

- 1. **Quasi-mapping**

Transcripts were quantified via the Salmon package (version 0.8.2) using the transcriptome-based quasi-mapping-based mode[18]. The transcript index used during quasi-mapping was derived from the UCSC refseq rn6.0 mRNA library available at the following link: <http://hgdownload.soe.ucsc.edu/goldenPath/rn6/bigZips/refMrna.fa.gz>.

Following library indexing, the following commands were used for quasi-mapping in accordance with the Salmon guidelines:
salmon quant -i /path_to/transcripts_index -l ISR -1 /path_to/lane1_1.fastq.gz /path_to/_lane2_1.fastq.gz /path_to/_lane3_1.fastq.gz -2 /path_to/lane1_2.fastq.gz /path_to/_lane2_2.fastq.gz /path_to/lane3_2.fastq.gz -o /path_to/transcripts_quant/SampleX --dumpEq -- posBias --gcBias --writeUnmappedNames

Data files were assigned alternative names to blind the experimenter during the relevant stages of the quasi-mapping analysis.

- 1. **RNAseq Differential Expression Analysis**

Following sample quantification, the data were imported into R and summarized at the gene-level using the 'tximport’ function (v1.6.0) as per the vignette[19]. A differential expression analysis of the gene counts for Wistar and preSHR samples was performed using the ‘DESeq2’ command in the R package DESeq2 (v1.18.1)[20]. The significance level for differential gene expression was accepted at the Benjamini-Hochberg adjusted p value p.adj<0.05. The ‘LFCshrink’ function was used to shrink log_2_ fold change after analysis, for visualization and ranking of genes, as per the DESeq2 vignette[20]. To assess the relevance of the observed differentially expressed genes, the significantly different transcripts at the Benjamini-Hochberg p. adj<0.05 level were analyzed using the Database for Annotation, Visualization and Integrated Discovery (DAVID v6.8) tool suite [21]. A Kyoto Encyclopedia of Genes and Genomes (KEGG)[22] analysis was performed to provide information about pathway mapping. The over-represented KEGG groups were filtered to exclude groups with <10 genes. A conservative EASE score (a modified Fisher Exact statistical test) was applied (p<0.01) to the KEGG analysis as per DAVID (v6.8) recommendations[21]. The final KEGG families were selected at the significant level p<0.05.

- 1. **Two-Step qRT-PCR**

TaqMan^®^ Gene Expression Master Mix (ThermoFisher) was added to each cDNA sample in addition to the selected primer conjugated to a FAM dye and nuclease-free H_2_O. cDNA samples or no reverse transcriptase controls were optimally diluted for qRT-PCR reactions. A 96-well plate with 20 μl samples (2-3 repeats) were run on a qRT-PCR thermocycler (ABI, PRISM). Temperatures were held at 50 °C (2 min) and 95 °C (10 min) before thermal cycling (40 cycles) under the following conditions 95 °C (15 s), 60 °C (1 min). The relative amount of each transcript was calculated using the comparative method (∆∆ C_T_, rat; ∆ C_T_, human)[23].

- 1. **Förster Resonance Energy Transfer (FRET)**

Sensor expressing stellate neurons were imaged on an inverted Nikon microscope connected to an OptoLED fluorescence imaging system (Cairn Research Ltd). Cells were imaged on a 40X or 60X oil-immersion objective and images were captured with a CoolSNAP HQ2 digital CCD camera (Photometrics). A dual-view beam splitter (DV2 multichannel imaging system, Photometrics) included the emission filters for cyan fluorescent protein (CFP, Et480/30M), yellow fluorescent protein (YFP, ET535/40M) with a dichroic mirror at 505 nm (505DCXR). During imaging, cells were excited at 430 nm for 100 ms every 15 s. Emission intensities for CFP and YFP were acquired using OptoFluor software (Cairn Research Ltd) at 480 nm and 535 nm and CFP/YFP intensity ratios were calculated. Background fluorescence was subtracted from all emission intensity ratios and the data were expressed as intensity per unit time. For all experiments, the baseline emission intensity was recorded and averaged over a 30 second period. Emission intensities were converted to FRET ratios (R) and expressed as a percentage FRET change (%) from baseline using the following equation: FRET Change = ΔR/R_0_. Where ∆R = R-R_0_; R_0_ = CFP/YFP intensity ratio at baseline (t = 0-30 seconds); R = CFP/YFP intensity recorded at any time-point in the experiment (t = seconds).

- 1. **Protein Extraction and Assay Protocols**

Protein was extracted from human stellate ganglia that were processed individually. The stellate samples were homogenized in ice-cold dPBS without Ca^2+^ or Mg^2+^ and the protein within the samples was normalized using a standard protein assay (BioRad DC). Enzyme immuno assays (EIA) or enzyme-linked immunosorbent assays (ELISA) were conducted to detect the presence of the following proteins of interest in human stellate ganglia: AGT (CSB-E08564h, Cusabio), renin (dren00, R&DSystems), AngII (RAB0010-1KT, Sigma), ACE2 (LS-F5886, LSBio), Ang1-7 (CSB-E14242h). Briefly, standards or samples (2-3 repeats) were incubated in 96-well plates and each assay was carried out as per the manufacturer’s instructions. The absorbance or fluorescence from each well was measured within 5 minutes at the appropriate wavelength and the background was subtracted from primary absorbance values (Infinite F500, Tecan). The expression of each relevant protein was quantified using a standard curve generated from the supplied standards (GraphPad Prism, v7).

- 1. **Expression of Angiotensin Synthesizing Enzymes and Precursors in Sympathetic Stellate Ganglia**

Protein was extracted from 12-to-14 stellates obtained from three- to four-week young Wistar or preSHR stellate ganglia. In alternative experiments, protein was extracted and pooled from 10-to-12 stellates from 12-to-16-week-old adult Wistar or SHR. Rat stellates were pooled within the respective age-groups to obtain adequate protein concentrations. The stellate samples were homogenized in ice-cold dPBS without Ca^2+^ or Mg^2+^ and the protein within the samples was normalized using a standard protein assay (BioRad DC). Enzyme immuno assays (EIA), enzyme-linked immunosorbent assays (ELISA). Enzyme immuno assays (EIA) or enzyme-linked immunosorbent assays (ELISA) were conducted to detect the presence of the following proteins of interest in rat stellate ganglia: angiotensinogen (AGT, OKCD01237, Aviva), AngII (RAB0010-1KT, Sigma), Ang1-7 (CSB-E14241r,). Briefly, standards or samples (2-3 repeats) were incubated in 96-well plates and each assay was carried out as per the manufacturer’s instructions. The absorbance or fluorescence from each well was measured within 5 minutes at the appropriate wavelength and the background was subtracted from primary absorbance values (Infinite F500, Tecan). The expression of each relevant protein was quantified using a standard curve generated from the supplied standards. The data was fit to a sigmoidal log_10_(molar concentration) curve and each of the sample values were interpolated (GraphPad Prism, v7).

- 1. **Statistical Analysis**

RNA-seq data were analyzed using the Salmon Quasi-mapping method in the statistical programme R, as described. Other data were imported into GraphPad Prism software (v7) for graphical representation. All data are expressed as mean ± SEM. The FRET data were analyzed using a two-way analysis of variance (ANOVA) and peak values analyzed using an unpaired two-tailed Student’s t-test. All data are expressed as the mean ± SEM. Statistical significance was accepted at p<0.05 unless otherwise described.

1. **Results**
   1. **Expression of Angiotensin Synthesizing Enzymes and Precursors in Sympathetic Stellate Ganglia**

The relative protein expression of Agt, AngII and Ang1-7 was quantified in rat stellate ganglia. To obtain adequate protein concentrations, 12-14 stellates from young rats or 10-12 stellates from adult rats were pooled in each stellate sample. Agt measured by ELISA was found to be highly expressed in young Wistar (16750 ± 3472 pg/ml, n=3 samples), young preSHR (14928 ± 1990 pg/ml, n=3 samples), adult Wistar (16924 ± 1312 pg/ml, n=5 samples) and adult SHR stellate ganglia (15071 ± 1572 pg/ml, n=6 samples). Angiotensin I (AngI) was not detectable by ELISA, as concentrations fell below the assay detection limit (data not shown). EIAs for AngII were conducted on stellate samples and quantified in young preSHR (423.5 pg/ml ± 14.65, n=3 samples), young Wistar (274.5 pg/ml ± 13.06, n=3), adult SHR (465.8 pg/ml ± 38.67; n=6) and adult Wistar stellate ganglia (282.4 pg/ml ± 19.31, n=6). Ang1-7 concentrations were also detected in preSHR ganglia (104.3 pg/ml ± 14.0, n=3 samples), young Wistar ganglia (40.2 pg/ml ± 13.9, n=3 samples) adult Wistar (42.8 pg/ml ± 4.9, n=3 samples) and adult SHR (53.6 pg/ml ± 2.7, n=3 samples). The data are expressed as mean ± SEM (supplement 1A).

S1A. The protein expression of Agt, AngII and Ang1-7 were identified in young Wistar (n=3, W), preSHR (n=3, S) adult Wistar (n=3-6, W) and adult SHR (n=3-6, S) stellate ganglia. Agt concentrations (~14,000-17,000 pg/ml), AngII concentrations (~140-180 pg/ml) and Ang1-7 concentrations (~400-1000 pg/ml) were observed. Each data point represents 10-12 pooled stellate samples. Data are represented as mean ± SEM.

- 1. **Cyan fluorescent protein and yellow fluorescent protein emissions**

The CFP and YFP emissions obtained during administration of Ang1-7 and AngII (supplement 2A) or in vehicle-controlled (Tyrode) experiments (supplement 2B) demonstrate FRET changes in response to angiotensin peptide administration and highlight the stability of the biosensor fluorophores in the vehicle-controlled experiments, No FRET changes to Tyrode were observed in vehicle controls (n=4, S2C).

| A | B |
| --- | --- |
| **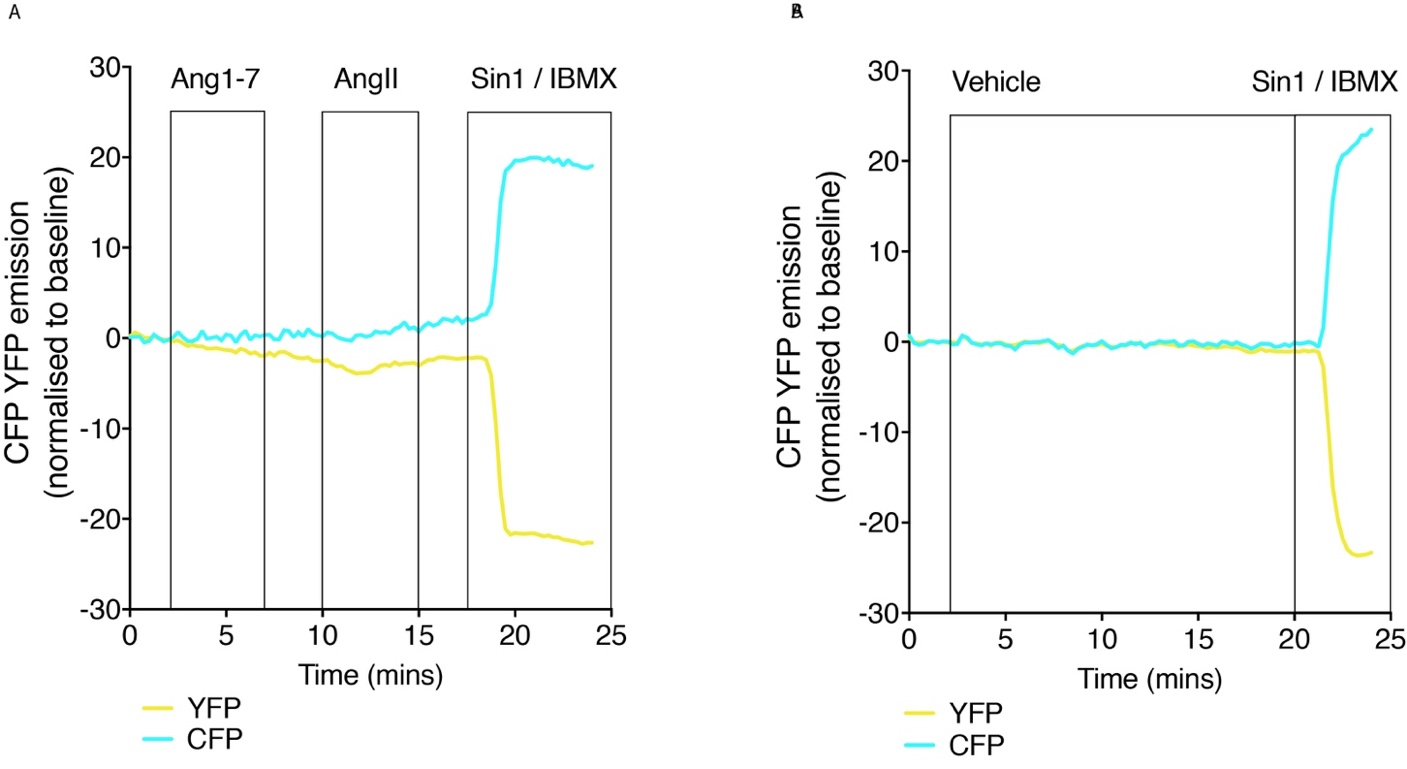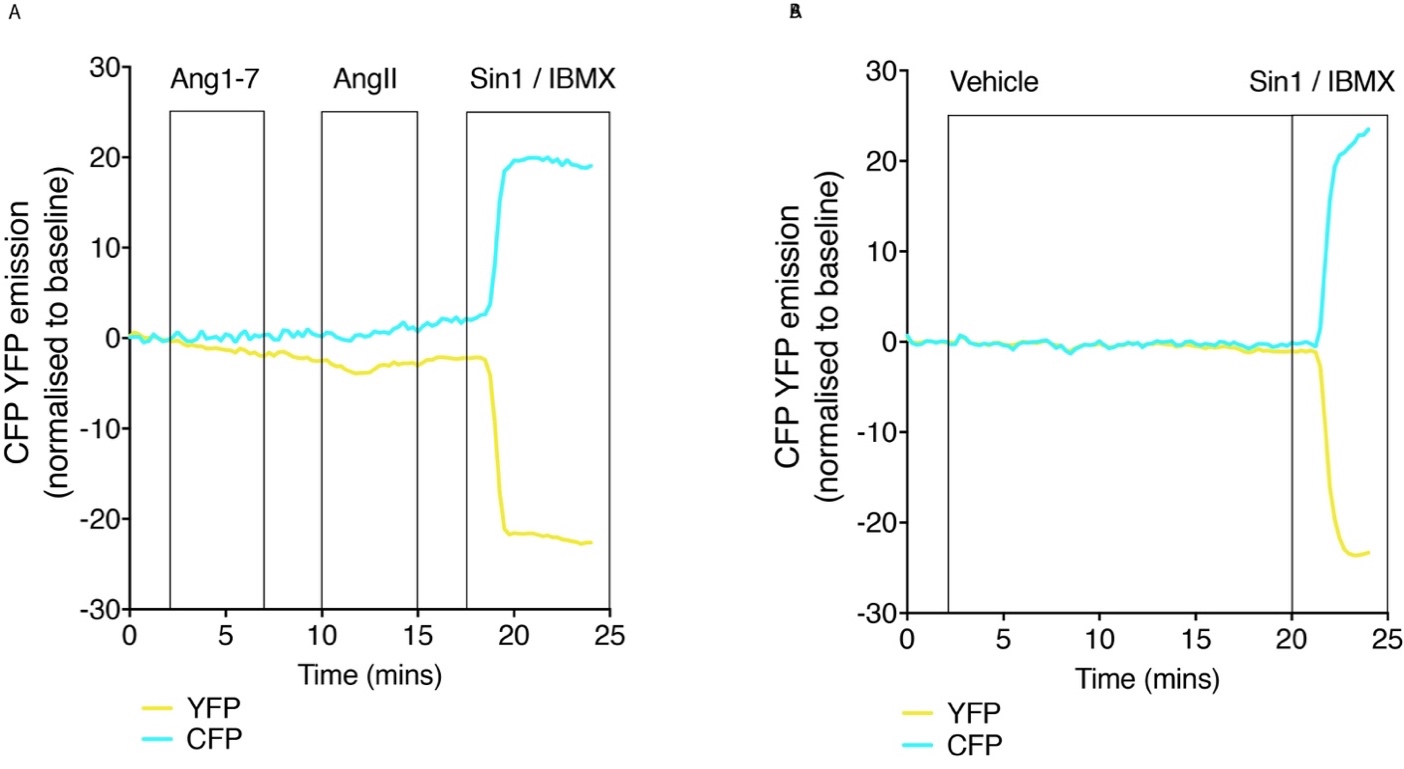** | |
| **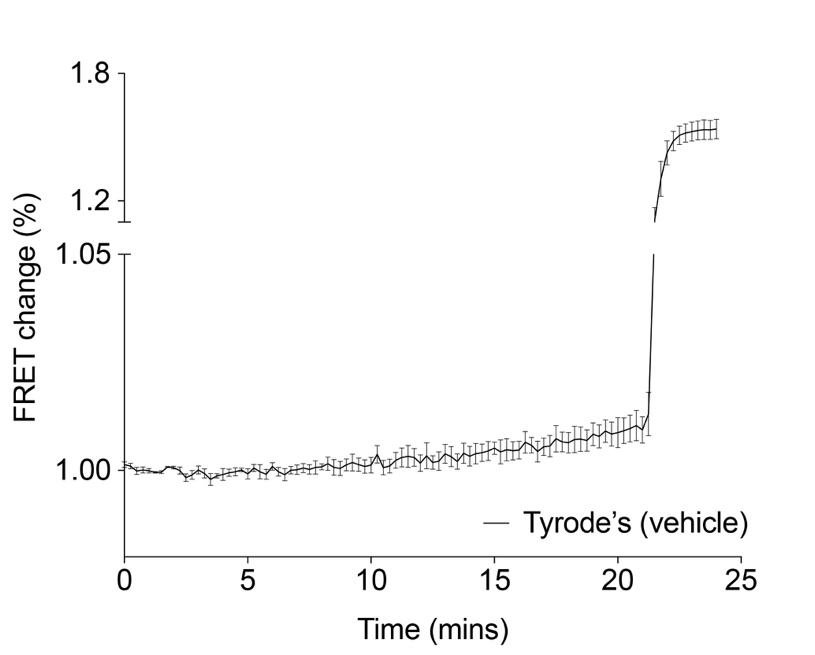** | |
| S2. Example CFP and YFP emission traces. CFP and YFP emissions were obtained during stimulation with Ang1-7 and AngII (2A) or in vehicle-controlled experiments during Tyrode administration (2B). No responses to Tyrode were observed in control experiments (n=4, 2C). Data are depicted as mean ± SEM. | |

**Conflict of Interest/Disclosure/s**

None.

**Acknowledgements**

We wish to acknowledge our collaborators Dr. Ajijola, Dr. Shivkumar and Dr. Ardell at UCLA for kindly extracting and shipping human sympathetic stellate ganglia from donor patients: 19, 21, 22, 23, 24. We thank Dr. Andrew Jefferson in the Micron Facility at the University of Oxford for his help and expertise using the Confocal microscope. We sincerely thank Peter M. Sloan for his assistance with the rat RNA extractions.

Funding

This project was funded by the Wellcome Trust OXION initiative (105409/Z/14/Z), British Heart Foundation Centre of Research Excellence and a BHF programme grant (RG/17/14/33085), and NIH SPARC (OT2OD023848) initiative.

**Author Contributions**

E.N.B and D.J.P planned the project. E.N.B. performed the experiments and analyzed the experimental data. O.N. carried out the neuronal culturing and FRET experiments. E.N.B. and D.J.P co-wrote the paper and edited the manuscript.

*All figures to be printed in colour

**References**

1. Judy WV, Watanabe AM, Murphy WR, Aprison BS, Yu PL. Sympathetic nerve activity and blood pressure in normotensive backcross rats genetically related to the spontaneously hypertensive rat. Hypertension 1979;1:598–604.

2. Ely DL, Friberg P, Nilsson H, Folkow B. Blood pressure and heart rate responses to mental stress in spontaneously hypertensive (SHB) and normotensive (WKY) rats on various sodium diets. Acta Physiol. Scand. 1985;123:159–69.

3. Shanks J, Manou-Stathopoulou S, Lu C-J, Li D, Paterson DJ, Herring N. Cardiac sympathetic dysfunction in the prehypertensive spontaneously hypertensive rat. Am. J. Physiol. Heart Circ. Physiol. 2013;305:H980–6.

4. Heijnen BF, Van Essen H, Schalkwijk CG, Janssen BJ, Struijker-Boudier HA. Renal inflammatory markers during the onset of hypertension in spontaneously hypertensive rats. Hypertens Res 2014;37:100–9.

5. Wilson AJ, Wang VY, Sands GB, Young AA, Nash MP, LeGrice IJ. Increased cardiac work provides a link between systemic hypertension and heart failure. Physiol Rep 2017;5.

6. Safar M, Chamiot-Clerc P, Dagher G, Renaud JF. Pulse pressure, endothelium function, and arterial stiffness in spontaneously hypertensive rats. Hypertension 2001;38:1416–21.

7. Doggrell SA, Brown L. Rat models of hypertension, cardiac hypertrophy and failure. Cardiovasc Res 1998;39:89–105.

8. Cunha RS, Dabiré H, Bezie I, Weiss AM, Chaouche-Teyara K, Laurent S, et al. Mechanical stress of the carotid artery at the early phase of spontaneous hypertension in rats. Hypertension 1997;29:992–8.

9. Sundaram A, Keah LS, Sirajudeen KNS, Singh HJ. Upregulation of catalase and downregulation of glutathione peroxidase activity in the kidney precede the development of hypertension in pre-hypertensive SHR. Hypertens Res 2013;36:213–8.

10. Smith TL, Hutchins PM. Central hemodynamics in the developmental stage of spontaneous hypertension in the unanesthetized rat. Hypertension 1979;1:508–17.

11. Dickhout JG, Lee RMKW. Structural and functional analysis of small arteries from young spontaneously hypertensive rats. Hypertension 1997;29:781–9.

12. Lee RM. Structural alterations of blood vessels in hypertensive rats. Can. J. Physiol. Pharmacol. 1987;65:1528–35.

13. Pijacka W, McBryde FD, Marvar PJ, Lincevicius GS, Abdala APL, Woodward L, et al. Carotid sinus denervation ameliorates renovascular hypertension in adult Wistar rats. J. Physiol. (Lond.) 2016;594:6255–66.

14. Oliveira-Sales EB, Colombari E, Abdala AP, Campos RR, Paton JFR. Sympathetic overactivity occurs before hypertension in the two-kidney, one-clip model. Exp. Physiol. 2016;101:67–80.

15. Abdala AP, McBryde FD, Marina N, Hendy EB, Engelman ZJ, Fudim M, et al. Hypertension is critically dependent on the carotid body input in the spontaneously hypertensive rat. J. Physiol. (Lond.) 2012;590:4269–77.

16. Bardsley EN, Davis H, Buckler KJ, Paterson DJ. Neurotransmitter switching coupled to β-adrenergic signaling in sympathetic neurons in prehypertensive states. Hypertension 2018;71:1226–38.

17. Conesa A, Madrigal P, Tarazona S, Gomez-Cabrero D, Cervera A, McPherson A, et al. A survey of best practices for RNA-seq data analysis. Genome Biol. 2017;:1–19.

18. Patro R, Duggal G, Love MI, Irizarry RA, Kingsford C. Salmon provides fast and bias-aware quantification of transcript expression. Nat. Rev. Cardiol 2017;14:417–9.

19. Soneson C, Love MI, Robinson MD. Differential analyses for RNA-seq: transcript-level estimates improve gene-level inferences. F1000Res 2015;4:1521.

20. Love MI, Huber W, Anders S. Moderated estimation of fold change and dispersion for RNA-seq data with DESeq2. Genome Biol. 2014;15:31–62.

21. Huang DW, Sherman BT, Lempicki RA. Systematic and integrative analysis of large gene lists using DAVID bioinformatics resources. Nat. Protoc. 2009;4:44–57.

22. Kanehisa M, Furumichi M, Tanabe M, Sato Y, Morishima K. KEGG: new perspectives on genomes, pathways, diseases and drugs. Nucleic Acids Res 2017;45:D353–61.

23. Schmittgen TD, Livak KJ. Analyzing real-time PCR data by the comparative CT method. Nat. Protoc. 2008;3:1101–8.
